# Prevention, Detection, and Management of Heart Failure in Patients Treated for Breast Cancer

**DOI:** 10.1007/s11897-020-00486-8

**Published:** 2020-09-26

**Authors:** Agneta Månsson Broberg, Jürgen Geisler, Suvi Tuohinen, Tanja Skytta, Þórdís Jóna Hrafnkelsdóttir, Kirsten Melgaard Nielsen, Elham Hedayati, Torbjørn Omland, Birgitte V. Offersen, Alexander R. Lyon, Geeta Gulati

**Affiliations:** 1 Department of Cardiology, Karolinska University Hospital, Stockholm, Sweden; 2 Department of Medicine, Huddinge, Karolinska Institutet, Stockholm, Sweden; 3 Department of Oncology, Akershus University Hospital, Lørenskog & Institute of Clinical Medicine, University of Oslo, Campus AHUS, Lørenskog, Norway; 4 Heart and Lung Center, Helsinki University Hospital, Helsinki, Finland; 5 Department of Oncology, Tampere University Hospital, Tampere, Finland; 6 Department of Cardiology, Landspitali University Hospital, Reykjavík, Iceland and Faculty of Medicine, University of Iceland, Reykjavík, Iceland; 7 Department of Cardiology, Aarhus University Hospital, Aarhus, Denmark; 8 Department of Oncology-Pathology, Karolinska Institute, Stockholm, Sweden; 9 Department of Breast Cancer, Sarcoma and Endocrine Tumors, Theme Cancer, Karolinska University Hospital, Stockholm, Sweden; 10 Department of Cardiology, Akershus University Hospital, Lørenskog and Institute of Clinical Medicine, University of Oslo, Oslo, Norway; 11 Department of Experimental Clinical Oncology & Department of Oncology, Aarhus University Hospital, Aarhus, Denmark; 12 Cardio-Oncology Service, Royal Brompton Hospital and Imperial College London, London, UK; 13 Department of Cardiology, Oslo University Hospital, Postbox 4950, Ullevål, Nydalen, 0424 Oslo, Norway; 14 Department of Research, Akershus University Hospital, Lørenskog and Institute of Clinical Medicine, University of Oslo, Oslo, Norway

**Keywords:** Breast cancer, Cardiotoxicity, Heart failure, Trastuzumab, Anthracyclines, Radiation therapy

## Abstract

**Purpose of Review:**

Long-term survival has increased significantly in breast cancer patients, and cardiovascular side effects are surpassing cancer-related mortality. We summarize risk factors, prevention strategies, detection, and management of cardiotoxicity, with focus on left ventricular dysfunction and heart failure, during breast cancer treatment.

**Recent Findings:**

Baseline treatment of cardiovascular risk factors is recommended. Anthracycline and trastuzumab treatment constitute a substantial risk of developing cardiotoxicity. There is growing evidence that this can be treated with beta blockers and angiotensin antagonists. Early detection of cardiotoxicity with cardiac imaging and circulating cardiovascular biomarkers is currently evaluated in clinical trials. Chest wall irradiation accelerates atherosclerotic processes and induces fibrosis. Immune checkpoint inhibitors require consideration for surveillance due to a small risk of severe myocarditis. Cyclin-dependent kinases4/6 inhibitors, cyclophosphamide, taxanes, tyrosine kinase inhibitors, and endocrine therapy have a lower-risk profile for cardiotoxicity.

**Summary:**

Preventive and management strategies to counteract cancer treatment–related left ventricular dysfunction or heart failure in breast cancer patients should include a comprehensive cardiovascular risk assessment and individual clinical evaluation. This should include both patient and treatment-related factors. Further clinical trials especially on early detection, cardioprevention, and management are urgently needed.

## Introduction

Breast cancer is the leading cause of cancer in women worldwide with a mean age standardized incidence of 46.3/100000, ranging from 25.9 in South East Asia to 94.2 in Australia and New Zeeland [1]. While the incidence is slightly increasing, the mortality is decreasing, likely due to the effective screening programs and evolving treatment.

Breast cancer treatment is based on the individual tumor’s expression of specific markers as estrogen receptor (ER), progesterone receptor (PR), human epidermal growth factor receptor (HER) 2,the proliferation marker Ki-67, and the extent of the disease. Combining surgery, chemotherapy, and radiation therapy with targeted therapy, the outcome has improved markedly over the past two decades and the average 5-year survival is currently approximately 87% [1]. The prolonged survival has led to an increased focus on the off-target-effects of breast cancer treatment. In fact, cardiovascular complications may surpass cancer-related mortality in a subgroup of patients [2]. Short-term acute left ventricular dysfunction, arrhythmia, or ischemic events can hinder patients from receiving life-saving cancer treatment. The patients are also at risk of developing long-term cardiovascular morbidity, especially decreased left ventricular function and heart failure. Heart failure is associated with reduced quality of life and increased mortality. In this article we summarize the clinically most relevant risk factors for cardiovascular complications during breast cancer therapy and discuss strategies for prevention, detection, and management of cardiotoxicity. We will focus on left ventricular dysfunction and heart failure.

## Commonly Used Chemotherapy

**Anthracyclines**, i.e., epirubicin and doxorubicin, are among the most widely used conventional chemotherapeutics established in breast cancer treatment. Anthracyclines inhibit deoxyribonucleic acid (DNA) synthesis and alters DNA transcription and replication by intercalating DNA and ribonucleic acid (RNA) strands and inhibiting topoisomerase IIα [3]. Anthracyclines also induce an iron-mediated oxidative stress that damages DNA, proteins, and lipids [4]. Cardiomyocytes and their mitochondria seem to be especially vulnerable to the damage via inhibition of topoisomerase IIβ and increased oxidative stress [5]. Dysregulation of epigenomic and transcriptomic responses have also been reported [6]. Importantly, cardiotoxicity is a clinically dose-limiting adverse effect of anthracyclines. In a retrospective report including three randomized studies (doxorubicin/placebo), decompensated congestive heart failure increased from 4.7% at 400 mg/m^2^ doxorubicin to 48% at doses of 650–800 mg/m^2^ [7]. About 50% of these patients had an absolute left ventricular ejection fraction (LVEF) drop of more than 30%. Importantly so, using sensitive echocardiographic methods such as global longitudinal strain (GLS), early signs of cardiotoxicity have been seen in doxorubicin doses as low as 150 mg/m^2^ [8]. Acute anthracycline-induced cardiotoxicity occurs within a week from treatment and is rare (< 5%). It resembles an acute toxic myocarditis with a histopathological cardiomyocyte damage, inflammatory infiltration, and interstitial edema [6, 9]. Often electrocardiographic (ECG) changes are present with brady- or tachy-arrhythmias and clinical symptoms as palpitations, dyspnea, chest pain, heart failure, syncope, or cardiac arrest. Chronic anthracycline-induced cardiotoxicity is histopathological typically characterized by vacuole formation, necrosis, myofibril dropout, and fibrosis [9]. By echocardiography this can be seen as a drop in LVEF or GLS. A decline in LVEF of ≥ 10 percentage to a value below 50% is widely accepted to be of significance [10]. Typical risk factors for anthracycline-induced cardiotoxicity are age < 5 or > 65 years, a family history of premature cardiovascular disease, arterial hypertension, diabetes mellitus, hypercholesterolemia, current myocardial disease, prior radiation to chest or mediastinum, smoking, and obesity [11, 12••].

In conclusion, anthracyclines are a major cause of cancer treatment-related cardiotoxicity in breast cancer patients.

**Cyclophosphamide** is an alkylating agent that in breast cancer patients usually is combined with anthracyclines and taxanes in the adjuvant setting. The cardiotoxicity is described as ECG changes, left ventricular dysfunction, and heart failure. There is conflicting evidence about the incidence, and numbers vary from < 1 to 28% [13]. In the standard adjuvant regimen for breast cancer 600 mg/m^2^, cyclophosphamide is given 3–4 times with 3-week interval. Recently, a dose-escalating phase III trial tested intensified treatment with cyclophosphamide, in myeloma patients, with doses up to 500 mg orally day 1, 8, 15, and 3 weekly cycles until maximum response or intolerance. In total, 4 patients (< 1%) had cardiac disorders, and this was not further specified [14]. In conclusion, the results support the low-risk profile for cardiac morbidity using cyclophosphamide in modern breast cancer treatment.

**Taxanes** are agents inhibiting mitotic activity, and the most frequently used drugs are docetaxel and paclitaxel. Taxanes can be used as monotherapy or combined with other chemotherapeutics including trastuzumab and pertuzumab. Cardiotoxicity with taxanes is infrequent. Reports are conflicting as the cardiotoxic definition is inconsistent, including asymptomatic bradycardia to ischemic events [15]. Additionally, it has been debated whether concomitant use of paclitaxel and doxorubicin aggravate doxorubicin induced cardiotoxicity [16, 17]. The incidence of docetaxel associated heart failure is < 2% [16, 18]. The incidence of myocardial ischemia has only been reported in the drugs summary of product characteristics (SPC) [19]. In a Cochrane review of taxanes for adjuvant treatment of early breast cancer twenty three studies included data on cardiotoxicity [20]. Pooled analysis showed small to no difference in cardiotoxicity of taxane compared with non-taxane-containing cancer treatment regimens (OR 0.87, 95% CI 0.56–1.33). Additionally, there was no difference in the risk of cardiotoxicity comparing those with or without anthracycline treatment (HR 1.27, 95% CI 0.88 to 1.84). In conclusion, the cardiotoxic risks of taxanes are low.

### Targeted Therapy

#### Endocrine therapy

Endocrine therapy is a corner stone in the treatment of hormone receptor positive breast cancer subtypes (ER/PR positive). In the adjuvant setting, endocrine therapy is given to selected patients for 5 to 10 years. Estrogen has indirect and direct effects on the cardiovascular system through the serum lipoprotein metabolism, coagulation, and fibrinolytic systems, and the antioxidative capacity. There have been concerns that estrogen inhibition in breast cancer treatment might mimic the postmenopausal effect on the cardiovascular system resulting in worsened cardiovascular outcome [21]. In breast cancer patients, antihormonal therapy is based on two major strategies, ER-modulators or inhibition of estrogen synthesis. Modulation of the ER is performed with selective ER modulators as tamoxifen, or estrogen receptor down-regulators as fulvestrant. The last two decades have been dominated by the introduction of aromatase inhibitors for postmenopausal women, inhibiting estrogen synthesis, i.e., with anastrozole, letrozole, and exemestane. Tamoxifen has shown to exert positive effects on the blood lipid profiles [22] and is believed to have a favorable cardiovascular effect [23], but its thromboembolic effect needs to be taken into consideration [24]. Aromatase inhibitors have been compared with placebo in several trials and do not seem to increase the risk for cardiovascular events in a significant manner [22]. However, these studies are not powered for cardiac events and frequently do not report specific cardiovascular adverse events. Aromatase inhibitors, when compared with tamoxifen, show an increased risk of arterial events as myocardial infarction and angina, which must be interpreted in the context of the protective effect of tamoxifen [22, 25]. Tamoxifen is primarily given to premenopausal women, while aromatase inhibitors of the third generation, i.e., anastrozole, letrozole and exemestane, are used in postmenopausal women.

Mammalian target of rapamycin (mTOR) inhibitors may be used in combination with exemestane in selected postmenopausal breast cancer patients with metastatic disease and have shown improved progression free survival compared with exemestane alone [26, 27]. mTORs can exhibit an indirect effect on cardiovascular health by metabolic changes, i.e., glucose and lipid metabolism, but are generally well tolerated [28]. Overall, the cardiovascular tolerability of endocrine therapy is high.

### HER2 Targeted Drugs

Approximately 15% of breast cancers are HER2 positive and are associated with aggressive behavior of the tumor [29]. Trastuzumab, a humanized monoclonal antibody against the extracellular domain of HER2, is in breast cancer often used in combination with chemotherapy or endocrine treatment [30, 31]. **Pertuzumab** is a recombinant humanized monoclonal antibody that binds to the extracellular domain of HER2 and functions as a dimerization inhibitor. As pertuzumab and trastuzumab binds to different domains of the HER2, a combination therapy is more beneficial than trastuzumab alone [32].

The HER2 receptor (also known as ERBB2 receptor) is expressed on cardiomyocytes, where it is involved in intracellular signaling pathways controlling apoptosis, cell regeneration, and to some extent the contractile function [33]. Blocking HER2 by trastuzumab may cause left ventricular dysfunction [32], but the complete mechanism of trastuzumab-induced cardiotoxicity is still not fully understood. In the clinical setting trastuzumab-induced myocardial dysfunction is potentiated by prior treatment with anthracyclines, which is also supported by preclinical studies [34–37]. Other risk factors include age > 65 years, high body mass index > 30 kg/m^2^, hypertension, and lower LVEF at baseline, while there are still conflicting data on other cardiovascular risk factors as diabetes, coronary artery disease, valvular dysfunction, and left ventricular hypertrophy [34, 38]. Trastuzumab-induced left ventricular dysfunction is to some extent reversible when paused or discontinued [10, 39–41]. Other studies have shown a persistent reduction in the left ventricular function after trastuzumab cessation [42–44]. In a study with 251 HER2 positive breast cancer patients, 42 patients had a LVEF drop of > 10% unit to a value below 50%, and 40% of these patients did not regain full cardiac function after enalapril and carvedilol treatment [42]. Whether this irreversibility is due to concomitant anthracycline toxicity is still unclear. The clinical presentation ranges from asymptomatic impaired left ventricular function to fulminant heart failure [32, 41]. In 2012 a Cochrane report based on randomized controlled trials of adjuvant therapy in breast cancer, the range of left ventricular dysfunction was between 7 and 34%. The rate of clinical severe heart failure (New York Heart Association (NYHA) class III or IV) ranged between 0 and 4% [45]. The risk is higher in older patients. In a register study identifying more than 45,000 patients over 67 years and treated with adjuvant therapy for early breast cancer, the adjusted 3 year incidence of heart failure or left ventricular dysfunction was 31% for those treated with trastuzumab alone, 42% for those treated with trastuzumab and anthracycline, and 18% for patients without adjuvant therapy [46]. Dual therapy with trastuzumab and pertuzumab does not increase the risk of cardiotoxicity compared with trastuzumab alone [47, 48].

The tyrosine kinase inhibitors **lapatinib and tucatinib** are approved for the treatment of HER2-positive, trastuzumab-resistant metastatic breast cancer [49, 50]. Neratinib is in some countries approved for extended adjuvant treatment [51]. In clinical trials the risk of cardiotoxicity of these drugs are lower than for trastuzumab; however, these trials included a low risk cohort [50, 52, 53] .

In conclusion, anti-HER2 therapy with trastuzumab is associated with significant risk of developing cardiotoxicity. There is no evidence of increased risk of cardiotoxicity when pertuzumab is added to trastuzumab.

### CDK 4/6 Inhibitors

Dysregulation of the cyclin-dependent kinases (CDK) 4/6–cyclin-D cell-cycle pathway induces a loss of mitotic regulation [54] and is associated with endocrine resistance in breast cancer [55]. Small-molecule CDK4/6-inhibitors as ribociclib, palbociclib, and abemaciclib have shown the ability to inhibit the growth of ER–positive breast cancer cells, act synergistically with antiestrogens, and reverse endocrine resistance [55–58]. CDK4/6 inhibitors with either aromatase inhibitors or antiestrogens are currently recommended as first-line treatment option for metastatic ER-positive and HER2-negative breast cancers [49]. Regarding cardiac safety, a range of different cardiovascular toxicities has been reported. Moderate QT prolongation has been observed with ribociclib, and ECG monitoring is advised [57]. In addition, the risk of venous thromboembolic events is increased compared with endocrine treatment only [55, 57]. In cell models and rodents, the CD4 activation pathway is important in early cardiomyocyte remodeling after myocardial infarction [59]; however, the clinical significance of this is unclear.

### ICI

The use of immune checkpoint inhibitors (ICIs) has vastly improved clinical outcomes in several cancer types during the last decade. However, in breast cancer patients the effect of current ICI regimens has been less beneficial [60]. Atetzolizumab and pembrolizumab have been approved for breast cancer treatment [61, 62]. As ICIs improve the activity of cytotoxic T-lymphocytes through blocking a major inhibitory pathway, these drugs may also cause a number of immune-related adverse events [63]. A feared but rare ICI-related adverse event is myocarditis, which may be associated with mortality rates up to 50% [64, 65]. Other rare ICI-mediated cardiovascular toxicities are pericarditis, heart block, acute coronary syndromes, Takotsubo syndrome, and during longer treatment non-inflammatory heart failure [66]. The use of ICI is expected to increase, and the cardiovascular complications will be an important issue for further surveillance.

### Radiation Therapy

The aim of radiation therapy may be curative, adjuvant, or palliative, and most patients receive radiation as part of a multimodal treatment strategy including surgery, chemotherapy, and targeted therapies. For most patients the gains of improved survival and organ-preservation outweigh the risks of normal tissue effects as fibrosis, necrosis, and pain. Serious life-threatening late effects as heart disease and secondary cancers are uncommon. Radiation-associated heart disease usually materializes more than a decade after treatment [67]. With improved cancer survival, more patients are at risk of suffering from radiation-associated heart disease.

Both the micro- and the macro- coronary circulation and the left sided cardiac valves can be affected. In breast cancer patients a dose–response relationship between radiation dose to the heart and risk of major coronary events or cardiac death was established. A relative risk of major coronary events or heart death increased by 7.4% per 1 Gy increases in mean heart dose. Importantly, there was no lower safe dose [68]. Pre-existing vascular disease may increase the risk of cardiac complications [69]. Radiation-associated heart disease has shown to be more lethal compared with heart disease not related to radiation [70]. Rarely, the radiation effect on the heart may occur acutely or subacutely (pericarditis and/or myocarditis), but usually, the effect occurs within 10 years in women with pre-existing coronary disease and 10+ years in lower risk individuals where healthy coronary vessels were exposed to radiation. Restrictive cardiomyopathy or constrictive pericarditis represents an advanced stage of damage due to fibrosis of the myocardium or pericardium with severe diastolic dysfunction and signs and symptoms of congestive heart failure [67, 71]. In conclusion radiation-induced cardiac disease may present decades after treatment completion.

### Detection

#### Imaging

Cardiac dysfunction secondary to cancer treatment can be defined as an absolute drop in LVEF of ≥ 10% to a value below < 50% [10]. Nuclear multigated acquisition (MUGA) and echocardiography are the most common modalities for serial LVEF measurements. A significant decline in LVEF might influence the continuation of cancer treatment; hence, LVEF measurements should be repeated after 2–3 weeks to confirm the decline [72]. The method of choice and vendor should preferably be consistent through the surveillance period and adapted to local availability and expertise (Table 1). LVEF comparison between different modalities is not advisable. If possible, MUGA should be avoided mainly due to radiation exposure [10]. Cardiac magnetic resonance imaging (CMR) is a reliable method for assessment of cardiac function and is the gold standard to measure volumes and EF. CMR adds value in differential tissue diagnostics; however, its unavailability and high cost precludes a more general use. As new cancer therapy-induced adverse effects arise, the role of CMR may become more prominent. ICI-mediated perimyocarditis might be missed by simple LVEF measurement; instead, it should be examined by CMR with T1 and T2 mapping to detect myocardial edema along with late gadolinium contrast enhancement of the inflamed pericardium and myocardium [73].

**Table 1:** Recommended imaging for detection and follow-up of cardiotoxicity in patients treated for breast cancer

Imaging modality	Method	Normal range	Detection of cardiotoxcity	Pro/con	Recommendation
Echo, 2D	LVEF, biplane Simpson	> 53%	≥ 10% absolute change to a value <50%	Widely accessible and used, but relatively high variability	Recommended in combination with GLS and biomarkers
Echo, speckle tracking	GLS	> 18%	value <18% or > 15% relative reduction from baseline	High reliability and validity, sensitive for early detection, especially in combination with biomarkers.	Recommended in combination with 2D echo and biomarkers
Echo, 3D	LVEF, 3D	> 55%	≥ 10% absolute change to a value < 50%	High reliability, not so widely used, more complicated than 2D	Recommended if available
CMR	LVEF	> 55%	≥ 10% absolute change to a value < 50%	Reliable method, low availability, add tissue information when needed	Recommended when tissue information is necessary (i.e., myocarditis)

*CMR* cardiac magnetic resonance imaging, *Echo* echocardiography, *GLS* global longitudinal strain, *LVEF* left ventricular ejection fraction

When using echocardiography, the most reliable measurement of LVEF is the transthoracic three-dimensional volumetric method. When not available, biplane Simpson method from two-dimensional echocardiography should be used. Two-dimensional GLS measured using speckle tracking is a more sensitive method than LVEF to evaluate early changes in left ventricular systolic function and should be used together with LVEF [10, 12•, 72]. It has been suggested that a GLS value of <18% or a relative reduction of 15% from baseline is significant [12•, 72]. However, these thresholds are based on small observational studies. It is unconfirmed if early change in GLS values will predict persistent decrease in LVEF or the development of systolic heart failure. Results from the SUCCOUR (Strain sUrveillance of Chemotherapy for improving Cardiovascular Outcomes) study, a randomized controlled international multicenter trial of GLS and EF in surveillance of cardiotoxicity in cancer patient, is highly awaited [74]. The target inclusion is 320 patients with a follow-up time of 3 years. The results will hopefully provide evidence for whether the use of GLS for surveillance for cardiotoxicity improves clinical outcomes.

Other sensitive methods to measure cardiotoxicity, as changes in right heart function, diastolic function, and changes unveiled during exercise, are in their infancy regarding to their reliability, usability and overall clinical relevance [12••, 72, 75, 76].

### Echocardiographic Follow-Up During Treatment with Anthracycline and Trastuzumab

Routine cardiac surveillance under anthracycline and HER2-treament has been recommended by both the cardiology and oncology working groups [10, 12••, 77••]. Expert consensus papers argue that baseline cardiac function should be assessed before adjuvant anthracycline treatment, especially as part of baseline risk assessment [10, 72, 76, 77••, 78].

### Anthracyclines

A baseline cardiac evaluation is desirable in all patients scheduled to receive anthracycline chemotherapy using the new Heart Failure Association of the European Society of Cardiology in collaboration with the International Cardio-Oncology Society (HFA-ICOS) baseline risk stratification proforma [79••]. Based on the accumulated dose-dependent toxicity of anthracyclines, follow-up is recommended, especially when cumulative doxorubicin-doses exceeds 240 mg/m^2^ [10, 77••]. These strategies have not been validated to prevent long-term cardiac events, nor has long-term follow-up after completion of anthracycline-therapy.

### Trastuzumab

For HER2-targeted therapies, a baseline measurement of cardiac function by LVEF and GLS is suggested using the new HFA-ICOS baseline risk stratification proforma [79••]. According to The Food and Drug Administration (FDA) approval, serial cardiac evaluation during trastuzumab treatment should be performed every 3rd month. This is based on clinical trial protocols. However, this ‘one size fits all’ approach is not logical clinically, and a more personalized monitoring schedule based upon baseline risk has been suggested [79••]. Even though frequent cardiac surveillance are recommended [12••], studies on the optimal frequency of cardiac monitoring during trastuzumab treatment are lacking [80].

### Biomarkers

Serum cardiac biomarkers indicating cardiac injury or dysfunction can be considered incorporated to the surveillance of patient at risk of cardiotoxicity. Even though a wide selection of different biomarkers has been studied, B-type natriuretic peptides (BNP) and cardiac troponins have been adopted to clinical practice. The value of BNP to detect cancer treatment related cardiotoxicity is limited by other variables that influence their levels including hemodynamic changes from stress, hypertension, infusions, vomiting, diarrhea, etc. A markedly elevated level of BNP has a high sensitivity for heart failure, while the specificity is variable [81]. It is unclear whether a rise in BNP precedes a decline in LVEF during breast cancer treatment [82]. Elevated levels of BNP during radiation therapy has been demonstrated, but the significance of this is unclear [83, 84]. While BNP is a marker of cardiac dysfunction, cardiac troponins are markers of myocardial injury. A rise in cardiac troponin I during high-dose anthracycline treatment has been a strong predictor of later LVEF decline and clinical events [85]. However, it is unclear if a rise in high sensitivity troponin precedes a decline in LVEF in contemporary breast cancer treatment [82]. Recently, the International CardioOncology Society-one trial (ICOS-ONE) evaluated the hypothesis of biomarker-guided cardioprotection versus primary prevention in 273 cancer patients scheduled to receive anthracycline chemotherapy, including breast cancer patients [86]. The results showed that the incidence of new troponin elevation was 23–26% in both arms. Enalapril pre-treatment in primary prevention did not prevent the troponin elevation, but it did reduce maladaptive remodeling and LV dysfunction in the very few individuals who sustained a cardiac injury and troponin rise. This trial was limited by relatively small numbers, variability of the troponin assays used in the recruitment hospitals, a relatively low risk cohort, and 14% of patients in the primary prevention arm stopping enalapril due to side effects.

Previous guidelines recommend monitoring troponin in high-risk patients to guide the decisiveness of cardioprotective management [10, 77••]. The most recent position statement from the HFA-ICOS recommends measurement at baseline pre-treatment to contribute to risk assessment [79••].

An increase in cardiac troponin is one of the diagnostic criteria for ICI-myocarditis [87]. Ideally, a cardiac troponin should be measured at baseline pre-treatment to place any future values in context. The benefit of routine cardiac troponin monitoring in all cancer patients receiving ICI without any cardiac symptoms is unclear.

## Preventive and Management Strategies

### Risk Factor Assessment

**Fig. 1:**
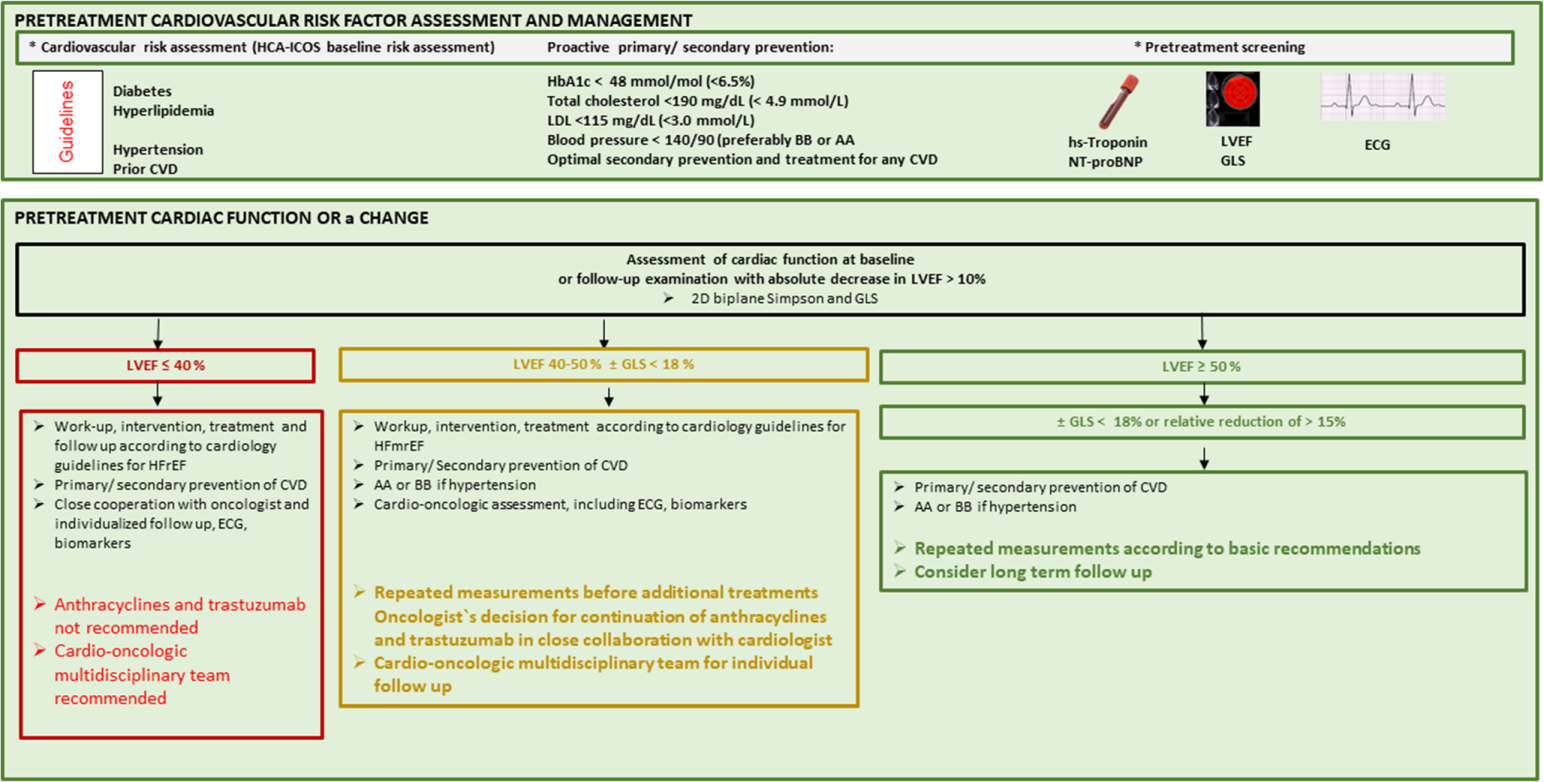
Detection and management of heart failure in patients receiving treatment for breast cancer. AA angiotensin antagonist, BB beta blocker, CVD cardiovascular disease, ECG electrocardiographic, GLS global longitudinal strain, HCA-ICOS Heart Failure Association of the European Society of Cardiology in collaboration HFmEF heart failure with midrange ejection fraction, HFrEF heart failure with reduced ejection fraction, Hs-Troponin high sensitive troponine with the International Cardio-Oncology Society, LDL low dense lipoprotein, LVEF left ventricular ejection fraction, NT-proBNP B-type natriuretic peptides

A pre-treatment cardiovascular risk assessment should be made for all patients using the new HFA-ICOS baseline risk stratification proformas to minimize the additional risk for developing cardiovascular disease during cancer treatment, i.e., evaluating baseline cardiac function, biomarkers, prior cardiovascular disease, and cardiovascular risk factors including glucose levels, lipid status, and checking blood pressure [79••]. These factors should be treated according to guidelines (Fig. 1). Beta blocker and/or an angiotensin antagonist should be considered for the treatment of hypertension as these drugs have shown additional cardioprotective effect during anthracyclines and/or trastuzumab treatment [88–94].

Patients with established cardiovascular disease should be on optimal treatment, and a recent echocardiogram is essential to verify their cardiac function. A pre-existing diagnosis of heart failure does not necessarily exclude treatment with anthracyclines or trastuzumab but identifies high-risk patients. A careful cardiac evaluation should determine the reason for the heart failure, i.e., heart failure with reduced (HFrEF) or preserved ejection fraction (HFpEF) or reversible causes, i.e., arrhythmia, coronary disease with ischemia, or Takotsubo syndrome. Smaller studies are indicating that even those with reduced or decline in LVEF may tolerate HER2-targeted therapy under cardioprotective treatment and close cardiac monitoring [41, 95•, 96•]; however, larger studies are awaited to confirm these findings.

### Management of Left Ventricular Dysfunction

In standard heart failure care and prevention there is strong evidence for symptom relief and mortality benefits for the treatment of HFrEF (LVEF < 40%) [97]. Breast cancer patients presenting with HFrEF should receive standard heart failure care according to guidelines. This includes both pharmacological treatment as angiotensin antagonists, beta blockers, mineral corticoid receptor antagonists (MRA), sacubitril and ivabradine, and device treatment [81]. One known side effect of MRA spironolactone is gynecomastia due to peripheral conversion of testosterone to estradiol. Several studies have studied the use of spironolactone, but there has been no evidence of increased risk for breast cancer [98]. There is no proven treatment for HFpEF (LVEF > 50%) and heart failure with midrange ejection fraction (HFmrEF) (LVEF between 40 and 50%). Although the evidence is not strong, new studies support continuation of Trastuzumab if LVEF is > 40% in asymptomatic patients [95•, 96•]. If LVEF drops ≥ 10% to a value < 50%, treatment with angiotensin antagonists and beta blockers are recommended [10, 77••, 95•, 96•]. Some early preventive studies during trastuzumab are more consistent with the protective effect of angiotensin antagonists than beta blockers [88–90, 92]. Data are lacking of when early cardioprotective treatment should be commenced, it should be evaluated based on change in cardiac function, elevated/rise in troponin and BNP, and cardiovascular comorbidities. Interestingly studies show that the use of beta blockers may attenuate the rise in cardiac troponin during anthracycline treatment, in none of these studies did beta blocker treatment effect the LVEF [90, 99]. The true meaning of mitigating the rise of troponin is unclear as long-term follow-up data is missing.

Dexrazoxane is well documented as a cardioprotective drug during anthracycline treatment [100]. Its use has been subdued because of concerns that it may either reduce the efficacy of anthracycline treatment against the primary malignancy or increase the risk of second malignancies. However, Cochrane Database Systemic Review analyses have shown no long-term safety concerns [100].

### Endocrine Treatment

Compared with placebo neither tamoxifen nor aromatase inhibitors increase the risk of myocardial infarction. There are no studies comparing discontinuation–continuation of endocrine therapy if a cardiovascular event occurs.

### Radiation Therapy

There is no evidence of cardioprotective treatment during radiation therapy. Complications should be treated according to general recommendations. However, it is important to keep in mind that irrradiation may also damage the microvasculature contributing to heart failure and that irrradiation often effects the proximal parts of the coronary arteries causing fibrotic lesions that are difficult to treat. Whether percutaneous coronary intervention (PCI) on these lesions is related to more complications is unclear [101, 102].

### Immune Checkpoint Inhibitors

In patients with ICI-myocarditis early treatment with high-dose intravenous methylprednisolone of 500–1000 mg i.v. for 3 days is recommended. If improvement is seen, a switch to oral prednisolone of 0.5–1 mg/kg with a weekly weaning schedule can be made [66, 103•]. Delaying high-dose steroids in cases of ICI-myocarditis increases the risk of major adverse cardiac events including mortality [103•]. If the myocarditis does not settle with high-dose methylprednisolone, second line immunosuppressive drugs should be considered including mycophenolate mofetil and possibly abatacept [104]. Plasmapheresis can also be considered [105••, 106]. In asymptomatic cases where elevated troponin levels are detected by screening, oral prednisolone of 2 mg/kg/day may be considered [77••, 105••].

## Conclusion

Modern breast cancer treatment is effective and has improved long-term survival. However, the long-term cardiovascular mortality and morbidity in these patients have also risen. The risk of left ventricular dysfunction and heart failure is substantially increased following treatment with anthracycline and trastuzumab. Chest wall radiation accelerates the atherosclerotic process and induces fibrosis. Immune checkpoint inhibitors are associated with fatal myocarditis. CDK4/6 inhibitors, cyclophosphamide, taxanes, tyrosine kinase inhibitors, and endocrine therapy have a lower-risk profile for cardiotoxicity.

Treatment of cardiovascular risk factors is recommended to avoid cardiotoxicity. Echocardiography and measuring circulating cardiovascular biomarkers such as troponins and BNP are useful as baseline markers and in evaluating the deleterious effect cancer treatment may have on the heart. The frequency of surveillance is based on expert consensus rather than randomized trials. HFA Cardio-Oncology Study Group has recently suggested a structured pathways for baseline risk assessment and surveillance. Beta blockers and angiotensin antagonists have shown promising results to prevent cardiotoxicity during anthracycline and trastuzumab treatment. Cardiopreventive techniques should be used in breast radiotherapy to minimize the cardiac radiation dose. If ICI-induced myocarditis is suspected, early intervention with high dose methyl-prednisolone has shown promising results.

Further clinical trials focusing on early detection, cardioprevention, and management are needed to elucidate the best strategies to prevent short- and long term development of left ventricular dysfunction and heart failure and thus promoting overall survival and quality of life.

## Abbreviations

BNP: B-type natriuretic peptides
CDK: Cyclin-dependent kinases
CMR: Cardiovascular magnetic resonance imaging
DNA: Deoxyribonucleic acid
ECG: Electrocardiogram
ER: Estrogen receptor
GLS: Global longitudinal strain
HER (ERBB): Epidermal growth factor receptor (Receptor tyrosine-protein kinase)
HFA-ICOS: Heart Failure Association of the European Society of Cardiology in collaboration with the International Cardio-Oncology Society
LVEF: Left ventricular ejection fraction
mTOR: Mammalian target of rapamycin
MUGA: Nuclear multigated acquisition
PR: Progesterone receptor
